# Deafferentation-Induced Redistribution of MMP-2, but Not of MMP-9, Depends on the Emergence of GAP-43 Positive Axons in the Adult Rat Cochlear Nucleus

**DOI:** 10.1155/2011/859359

**Published:** 2011-10-23

**Authors:** Michaela Fredrich, Robert-Benjamin Illing

**Affiliations:** Neurobiological Research Laboratory, Department of Otorhinolaryngology, University of Freiburg, Killianst. 5, D-79106 Freiburg, Germany

## Abstract

The matrix metalloproteinases MMP-9 and MMP-2, major modulators of the extracellular matrix (ECM), were changed in amount and distribution in the rat anteroventral cochlear nucleus (AVCN) following its sensory deafferentation by cochlear ablation. To determine what causal relationships exist between the redistribution of MMP-9 and MMP-2 and deafferentation-induced reinnervation, kainic acid was stereotaxically injected into the ventral nucleus of the trapezoid body (VNTB) prior to cochlear ablation, killing cells that deliver the growth associated protein 43 (GAP-43) into AVCN. Deafferentation-induced changes in the pattern of MMP-9 staining remained unaffected by VNTB lesions. By contrast, changes in the distribution of MMP-2 normally evoked by sensory deafferentation were reversed if GAP-43 positive axons were prevented to grow in AVCN. In conclusion, GAP-43-containing axons emerging in AVCN after cochlear ablation seem to be causal for the maintenance of MMP-2-mediated ECM remodeling.

## 1. Introduction

Cochlear ablation entails Wallerian degeneration of the auditory nerve fibers and loss of their synaptic terminals in the cochlear nucleus (CN) [1]. This lesion-induced detrimental phase proceeds for some days, leaving the CN with a massively reduced input of excitatory afferents [2, 3]. Subsequent to the removal of degenerating axons and synaptic endings, a constructive phase of tissue reorganization is initiated, apparently comprising nervous regeneration and reinnervation [4, 5]. 

The growth-associated protein 43 (GAP-43) is a marker of axonal growth and synaptogenesis in the central nervous system (CNS) [6]. Highly expressed during early brain ontogeny, GAP-43 expression is turned down with the progression of postnatal development [6, 7] but stays high in some cerebral regions or may rise again if network modifications and synaptic remodeling are required [8, 9]. Following sensory deafferentation, GAP-43 reemerges in fibers and presynaptic terminals growing into the anteroventral CN (AVCN) [10, 11]. These fibers originate from neurons of the medial olivocochlear (MOC) system arising in the ventral nucleus of the trapezoid body (VNTB), a rhombencephalic region characterized by conspicuously large cholinergic neurons [10, 12]. On their way to the inner ear, MOC neurons give off axon collaterals into the cochlear nucleus [13–15], terminating in the marginally located granule cell layer of AVCN in normal animals [16, 17]. These axon collaterals sprout into central parts of AVCN upon cochlear ablation, replacing the lost excitatory input of the auditory nerve [12]. In the present study GAP-43 was used as a marker for axonal growth and reactive synaptogenesis.

Nerve degeneration and regeneration entail and require extensive tissue dynamics which includes perishing of some structural elements and formation of others, disappearance of some molecular components and rise of others, and the movement of membranes, organelles, and molecules, all affecting different types of cells and the extracellular matrix (ECM). Matrix metalloproteinases (MMPs) are key modulators of the ECM in nerve tissue. They constitute a large family of mainly extracellularly operating enzymes [18] (for exceptions see [19, 20]). They are synthesized and secreted as inactive proenzymes and activated in pericellular regions [17, 18] to perform essential functions in neuroplasticity and the processes of Wallerian degeneration as well as axonal growth and regeneration (for review see [21–23]). Besides serving molecular signaling through the processing of ligands that then bind to specific cell surface receptors, MMPs also function in the physical restructuring of the pericellular environment [24]. 

The gelatinases MMP-9 and MMP-2 belong to the most abundant MMPs within the brain [25]. In an earlier study we were able to show a spatiotemporal relationship of MMP-2 accumulation in the neuropil with the emergence of GAP-43-positive nerve fibers and boutons in the sensory-deafferented AVCN [26] and suggested MMP-2 to be involved in the compensatory restructuring of neuronal networks that have suffered a massive loss of synaptic contacts. The same cannot be claimed of MMP-9. According to the literature, MMP-9 is often associated with early tissue responses due to neurodegeneration and related events following injury like neuronal death [27–29], glial scar formation [30], and opening of the blood brain-barrier [29].

With the present study, we aimed to settle two issues. First, we charted the staining pattern of MMP-9 and MMP-2 in AVCN at different points in time following ablation of the cochlea in order to see if their amount, distribution, or both are changed as a consequence of sensory deafferentation. Since we quickly noticed that little changes occurred in global staining for either MMP (manuscript in preparation), but that local changes centered around neuronal cell bodies were obvious, we here focus on the MMP staining of neurons and their surround. Second, we attempted to determine if there is a causal relationship between the deafferentation-induced redistribution of the MMPs and reinnervation of AVCN. We therefore lesioned VNTB prior to sensory deafferentation to prevent axonal sprouting and synaptogenesis in AVCN [12]. Deviations from the normal deafferentation-induced redistributions of MMP-9 and MMP-2 were documented. 

## 2. Experimental Procedures

### 2.1. Animals

Brains of 34 female Wistar rats of ages 6 to 10 weeks were used in this study. Care and use of the animals as reported here were approved by the appropriate agency (Regierungspräsidium Freiburg, Germany, permission number 35/9185.81/G-07/22). For surgery, rats were deeply anesthetized with an intraperitoneal injection of a mixture of ketamine (50 mg/kg, Ketanest, Parke-Davis, Ann Arbor, Mich, USA) and xylazine (5 mg/kg, Rompun, Bayer Leverkusen, Germany). Animals were divided into 4 experimental groups (Table 1). Group 1 consisted of control animals that did not receive any surgery. Group 2 included animals receiving injections of phosphate buffered saline (PBS) into VNTB and animals unilaterally injected with kainic acid (KA) into VNTB. Animals in group 3 received a unilateral cochlear ablation and survived for 1, 3, or 7 postoperative days (PODs). Group 4 consisted of animals receiving KA injections into VNTB on the right side of the brainstem followed by cochlear ablation on the left side 1 to 3 weeks later. After cochlear ablation, animals survived for 3 or 7 days (Table 1). Investigations at POD1 were not done in this group because GAP-43 does not notably emerge in AVCN before POD3 [4]. Throughout this study, the side of the brain on which the cochlear ablation was done is referred to as ipsilateral. 

### 2.2. Cochlear Ablation

Following anesthesia (see above), tympanic membranes were checked for integrity and transparency. Unilateral cochlear ablation was performed on the left side using a retroauricular approach [4]. After the facial nerve was sectioned at its exit from the skull, tympanic membrane and ossicles were removed and the opening in the bulla tympani was widened to provide good visibility of the cochlea. The bony wall of the cochlea was opened with a diamond drill and the interior of the cochlea, including the spiral ganglion, was completely removed. Cochlea and bulla were filled with Gelfoam (Marbagelan/Spongostan, Ferrosan AS, Soeborg, Denmark) and the wound was surgically closed.

### 2.3. Stereotaxic Injection of KA into VNTB

Animals were positioned in a stereotaxic head holder. A craniotomy was made with a diameter of approximately 3 mm and the dura mater opened. The hole was centered at 2 mm caudal and 2 mm lateral to point lambda [31]. A Hamilton syringe connected to a glass micropipette with a tip diameter of approximately 50 *μ*m was used for unilateral (right) pressure injection of KA (kainic acid monohydrate, Sigma-Aldrich, Taufkirchen, Germany, K0250-10MG, 100 nL per injection, 1 mg/100 *μ*L Locke's solution). Injections were centered in VNTB with the stereotaxic target coordinates 1.8 mm lateral, 0.7 mm ventral, and 0.3 mm rostral to interaural zero in rats with a body weight of approximately 200 g. The injection lasted for 5 min and the pipette was left in position for another 5 min before withdrawal. The skull opening was covered with Gelfoam, and the scalp was surgically closed. Rats recovered from the injection before cochlear ablation on the opposite (left) side or direct histological processing of their brains. Bilateral injections of KA were not done for two reasons. First, the crossed projection of MOC neurons residing in VNTB dominates the uncrossed projection [13]. Correspondingly, experiments with contralateral KA injection followed by cochlear ablation showed a massive reduction of cochlear ablation-induced GAP-43 expression in the AVCN [12]. Second, rats receiving bilateral KA injections into the rhombencephalon large enough to cover VNTB have a low chance of survival [12].

### 2.4. Immunohistochemistry

Animals received a lethal intraperitoneal dose of Trapanal (50 mg in 1 mL distilled water/200 g body weight, Nycomed GmbH, Konstanz, Germany) and were perfused transcardially with a fixation solution containing 4% paraformaldehyde and 0.1% glutaraldehyde in 0.1 M phosphate buffer at pH 7.4. Brains were removed from the skull and cut into 40 *μ*m thick frontal sections on a microtome with vibrating blade (Leica VT 1000S, Benzheim, Germany) and the sections collected in 0.37% glycine dissolved in 0.1 M phosphate buffer at pH 7.4. Primary antibodies used in all experimental groups were raised against GAP-43, MMP-9, or MMP-2 (Table 2).

Different immunocytochemical procedures were employed. For visualizing GAP-43 in AVCN, free-floating sections were preincubated in 0.1% Triton X-100, 0.15% H_2_O_2_ and 5% normal serum, all in 0.02 M PBS at pH 7.4 for 30 min at room temperature. For MMP-2 and MMP-9 staining, the same preincubation steps were employed, but Triton X-100 was replaced by dimethylsulfoxide applied in 4 increasing concentrations of 5, 10, 20, and 40%. Compared to Triton X-100, dimethylsulfoxide does not penetrate as deeply into the sections, providing two clearly separable focal planes for quantitative particle detection. Sections were incubated with primary antibodies (Table 2) over night at 4°C. Incubation with the matching secondary antibodies (Table 2) and formation of an avidin-biotin complex (Elite-ABC; Vector Laboratories, Burlingame, USA) took 1 h each and were followed by staining with 0.05% 3.3-diaminobenzidine tetrahydrochloride (DAB; Sigma, Taufkirchen, Germany) and 0.005% H_2_O_2_ in 50 mM Tris buffer at pH 7.2. For staining of GAP-43 immunoreactivity, 0.3% ammonium nickel sulphate were added to the DAB solution. Sections stained for GAP-43 were mounted on gelatin-subbed slides, air dried, dehydrated in increasing grades of alcohol, and cover-slipped with Entellan (Merck, Darmstadt, Germany). Sections stained for MMP-9 and MMP-2 were incubated in 0.1% osmium tetroxide in cacodylic acid buffer at pH 7.2 for 4–7 min. Sections were then dehydrated in increasing grades of ethanol and flat-embedded in epon (Embed-812, Science Services, München, Germany), supporting morphological integrity. After hardening of the resin, areas of interest were photographed and selected for further analyses under the light microscope. Sections were reembedded in gelatin capsules and cut in a transverse plane into 0.5 *μ*m thick semithin sections using an ultramicrotome (Reichert-Jung Ultracut, Vienna, Austria). These sections were mounted on glass slides, air dried, and cover-slipped with Glycergel (Dako, Carpinteria, Calif, USA). 

**Figure 1:**
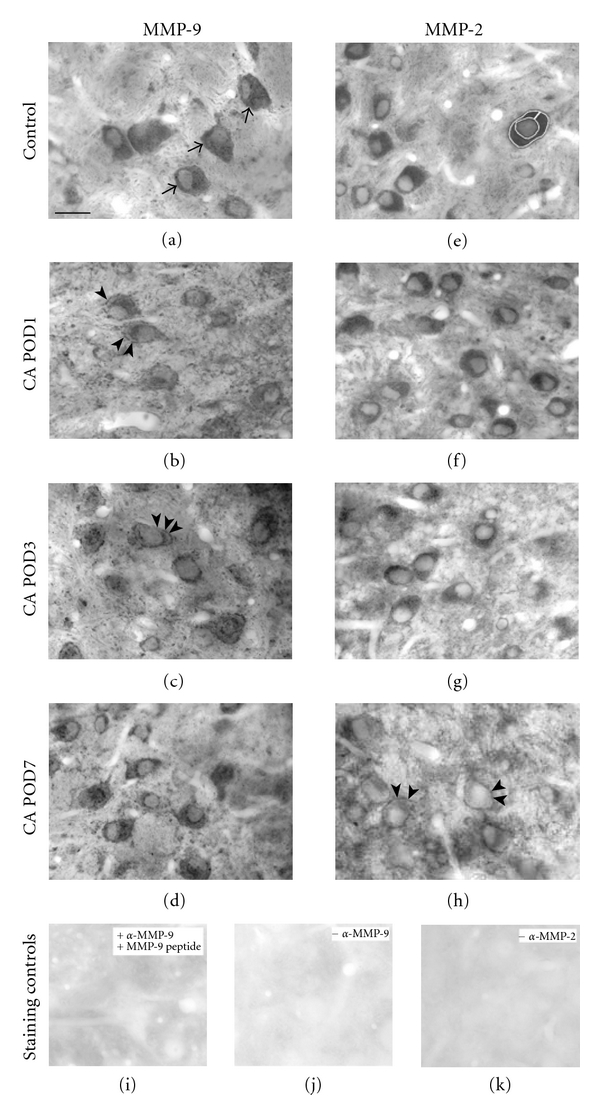
Time course and distribution of MMP-9 and MMP-2 realignments in AVCN on the side of cochlear ablation (CA), shown in 40 *μ*m thick sections stained with DAB and flat embedded in epon. (a) In AVCN of control animals, numerous neurons were strongly immunoreactive for MMP-9 in their cytoplasm (arrows). (b and c) By POD1 and POD3, MMP-9 immunoreactivity decreased in the cytoplasm of most neuronal somata. Beaded collars of MMP-9 immunoreactive particles emerged close to the plasma membrane (arrowheads). (d) By POD7, the pattern of MMP-9 staining has almost returned to control level (a). (e) MMP-2 was strongly expressed in the cytoplasm of AVCN neurons in control animals. The white line indicated around the cytoplasm of one neuron shows the sample field for quantitative analysis of MMP-staining intensity in neuronal cytoplasm used to obtain the data shown in Figures 6 and 7(e). (f) By POD1, only marginal changes in MMP-2 staining were seen. A derangement seen in tissue texture is caused by disintegrating auditory nerve fibers. (g) By POD3, the content of MMP-2 in cytoplasm of AVCN neurons slightly decreased. (h) This decrease continued towards POD7 when beaded collars of MMP-2 positive particles became visible in direct vicinity to the cell's plasma membrane (arrowheads). (i) and (h) Neutralization of the primary antibody prior to the staining procedure (i) and omission of the primary antibody against MMP-9 (j) verified specificity of the immunostaining. (k) Omitting incubation with antibody raised against MMP-2 resulted in failure of staining. Global contrast adjustment was identical for all photographs in this figure. Scale bar: 20 *μ*m.

To verify the specificity of all primary antibodies used, negative controls were run by omitting the primary antibodies in the corresponding incubation step (Figures 1(j), 1(k), and 4(a) inset). Specificity of the antibody raised against MMP-9 was additionally verified by preincubation with MMP-9 peptide for 2 hours at room temperature before exposing sections for immunohistochemical staining as described before. An equivalent product was unavailable for MMP-2. The failure of staining was equally obvious after these embedded sections were cut to be analyzed as semithin sections.

### 2.5. Nissl Staining

Brain sections destined for Nissl staining were washed in 0.02 M PBS at pH 7.4, mounted on gelatin-coated glass slides, and air dried. They were then defatted in alcohol, incubated in cresyl violet (Chroma, Stuttgart, Germany, 1A396; 1% in distilled water) for 2 min, dehydrated in increasing grades of ethanol, cleared in xylene, and cover slipped with Entellan.

### 2.6. Quantitative Data Acquisition

Photographs were taken through a ×5 or a ×100 objective with a digital camera (AxioCam, Zeiss, Jena, Germany) at 8 bit gray tone depth. For each type of staining, incubation parameters, microscope settings, exposure times, and global rendering of the photographs were held strictly constant across all experiments.

We determined the overall staining level of GAP-43 immunoreactivity in AVCN by gray tone means in ×5 photographs (Figure 4) using Photoshop CS (Ver. 8, Adobe, San Jose, Calif, USA). Analysis of heterogeneous GAP-43 staining was achieved by measuring gray tone means in two fields of equal size, placed in AVCN so that one field lied in the region with the faintest staining and the other in the region with the strongest staining (Figure 4(f)), avoiding marginal zones of AVCN. In sections showing homogeneously distributed GAP-43 immunoreactivity across AVCN and in controls (Figures 4(a)–4(e)) test fields where centered in the dorsal and ventral half of AVCN, respectively. Staining intensities were determined in AVCN on both sides of the brainstem and left-to-right or ipsilateral-to-contralateral ratios or ratios for paired test fields within the ipsilateral AVCN were calculated. 

**Figure 2:**
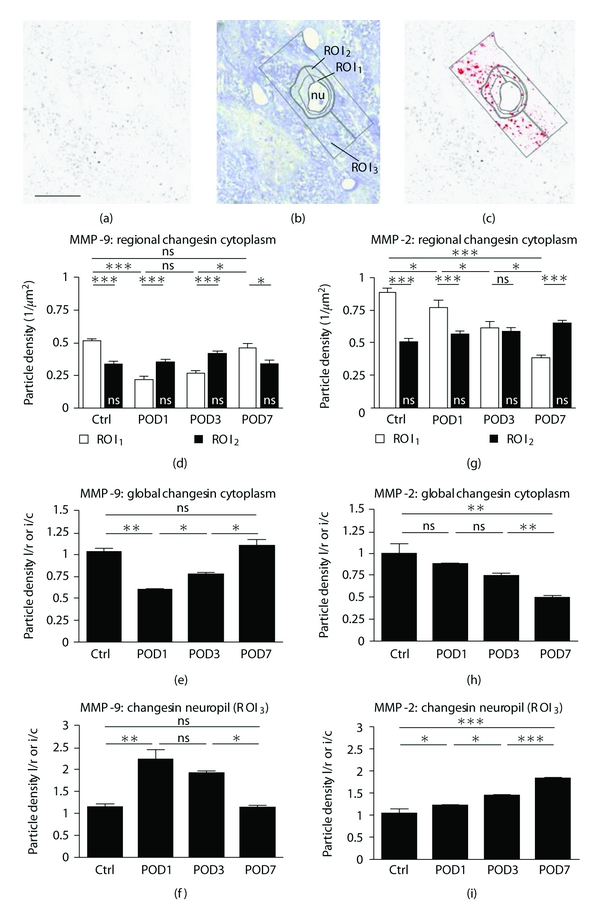
Semithin sections through AVCN stained for MMP-9 and MMP-2 immunoreactivity following cochlear ablation, and their quantitative evaluation. (a) By POD7, MMP-2 immunoreactivity showed up as particles. (b) Counterstaining of the same section with methylene blue, visualizing cellular boundaries. Lines indicate the regions of interest (ROI) sparing the cell nucleus (nu). (c) Same section as in (a) with the superimposed ROIs. Particles detected by computer-aided image analysis are indicated in red. Scale bar for (a)–(c) 20 *μ*m. (d) Subcellular redistribution of MMP-9 immunoreactive particles in AVCN neurons in control animals (ctrl) and at POD1, 3 and 7 following cochlear ablation. Bars indicate particle density in ROI_1_ and ROI_2_. ns designates insignificant changes within ROI_2_ at any time. (e), (f) Changes in MMP-9 particle density in cytoplasm (e) and neuropil (f) of AVCN neurons in controls (ctrl) and by POD1, 3 and 7 following cochlear ablation. Bars indicate left-to-right (l/r) or ipsilateral-to-contralateral (i/c) ratio of particle density. (g) Subcellular redistribution of MMP-2 immunoreactive particles within the cytoplasm of AVCN neurons in control animals (ctrl) and by POD1, 3 and 7. (h), (i) Changes in MMP-2 particle density in cytoplasm (h) and neuropil (i) of AVCN neurons in control animals (ctrl) and by POD1, 3 and 7 following cochlear ablation.

For MMP-9 and MMP-2, two different strategies for quantitative data acquisition were followed. In the first, densities of immunoreactive particles in neuronal cytoplasm and neuropil of AVCN were detected with analySIS (Soft Imaging System GmbH, Münster, Germany), setting thresholds of gray tone detection rather high and strictly constant for every ×100 photograph of semithin sections stained for MMP-9 or MMP-2, respectively (Figure 2(a)). Semithin sections contain immunoreactive particles in only one focal plane, allowing their unimpaired quantification (Figure 2(c)). After photographic documentation of the first 1 to 3 semithin sections cut (Figure 2(a)), cover-slips were removed and the sections counterstained with methylene blue-azure (1% azure II in distilled water and 1% methylene blue (Merck, Darmstadt, Germany, 9211 and 6045, resp.) in borax, mixed to equal parts) to visualize boundaries of cell bodies and nuclei (Figure 2(b)). Sections were then cover-slipped with Entellan and photographs of the same locations as already documented were taken. It was then possible to outline 3 regions of interest (ROIs), aiming to detect migrations of MMP-9 and MMP-2 positive particles from the cytoplasm into the surrounding neuropil of AVCN neurons, if they occur. The first ROI outlined the cytoplasm of neurons near their cell nucleus (ROI_1_). The second ROI outlined the cytoplasm near the plasma membrane (ROI_2_). If intracellular movements of MMPs towards the plasma membrane occurred, we should be able to detect them by comparing staining in these ROIs at different postoperative points in time. A third ROI (ROI_3_) was positioned to cover the neuropil immediately surrounding neuronal somata (Figure 2(b)) to determine if there is an increase of the respective MMP in pericellular regions over postlesional time. ROI_3_ was oriented along the cutting edge of the sections to prevent errors in particle detection due to decreasing DAB-staining intensity in section depths beyond reach of primary antibody diffusion (Figures 2(b) and 2(c)). Neurons were distinguished from glia by size and morphology. For statistical data acquisition, all neurons with clearly visible nucleus and cell boundary were taken into account. This study does not distinguish between different types of neurons. ROIs identified after counterstaining were assigned to the appropriate photographs of semithin sections and used to determine staining density of MMP-9 and MMP-2 immunoreactivity. Left-to-right or ipsilateral-to-contralateral ratios of particle densities in AVCN were calculated. 

Knowing that MMP-9 and MMP-2 particle density in semithin sections significantly decreased in the cytoplasm of AVCN neurons following deafferentation (Figure 2), we used the staining intensity of MMP-9 and MMP-2 in the cytoplasm of AVCN neurons in 40 *μ*m thick flat-embedded sections to identify possible transcellular movements of either MMP due to deafferentation. In the second strategy for quantitative data acquisition gray tone means were determined in the cytoplasm of AVCN neurons stained for MMP-9 or MMP-2 (Figure 1(e)) in photographs of flat-embedded sections taken with an ×100 objective. Left-to-right or ipsilateral-to-contralateral ratios of staining intensities (Figure 6) and the ratios of differently stained fields within the ipsilateral AVCN (MMP-2 only) were calculated (Figure 7). 

Statistical analysis was done using Prism4 (GraphPad 554 Software, Inc., La Jolla, Calif, USA). Significant differences between groups of measurements were determined by applying one-way analysis of variance (ANOVA) followed by Newman-Keuls post test or, if applicable, with two-tailed Student's *t*-test. Significance levels were indicated as ***P < 0.001, **P < 0.01, or *P < 0.05. Significance levels indicated by # in the figures identify significant differences against the control level. 

## 3. Results

### 3.1. Time Course of MMP-9 Realignments in AVCN Following Cochlear Ablation

In AVCN of the normal adult rat, MMP-9 expression was high in the cytoplasm of neuronal cell bodies (Figure 1(a)). Levels of MMP-9 were lower in the neuropil, resulting in their distinct appearance (Figure 1(a)). Following cochlear ablation, MMP-9 expression decreased in many neuronal somata as soon as by POD1 (Figure 1(b)). At the same time, numerous small MMP-9 immunoreactive particles emerged as beaded collars around the neuronal plasma membrane (Figure 1(b)). Whereas these effects were strong by POD1 and POD3 (Figures 1(b) and 1(c)), the pattern of MMP-9 staining almost returned to the preoperative condition by POD7 (Figure 1(d)). Despite the massive derangement of the tissue caused by auditory nerve degeneration, the overall staining density in these flat-embedded sections remained largely unchanged during the postoperative period examined (Figures 1(a)–1(d)). Negative controls of osmicated, flat-embedded sections showed now staining at all (Figure 1(j)), neither did sections incubated with the neutralized primary antibodies (Figure 1(i)).

To test the hypothesis that MMP-9 undergoes redistribution rather than a locally varying degradation and reexpression, we determined the density of MMP-9 immunoreactivity in semithin sections through AVCN quantitatively (Figures 2(a)–2(c)). In sections of control tissue, MMP-9 was found to be unevenly distributed in the cytoplasm of neurons (Figure 2(d)). The majority of MMP-9 immunoreactive particles was located in ROI_1_ near the cell nucleus, while the rest was located in ROI_2_ close to the plasma membrane (Figure 2(d)). This ratio was inverted by POD1 (Figure 2(d)) due to a significant decrease of the particle density in ROI_1_. Particle density in ROI_2_ did not change (Figure 2(d)). Measuring the density in the entire cytoplasm, we found a strong net decrease of MMP-9 immunoreactive particles by POD1 (Figure 2(e)) compared to control levels (Figure 2(e)). At the same time, MMP-9 particle density rose significantly in the neuropil surrounding the neurons (Figure 2(f)). By POD3, the intracellular distribution of MMP-9 seen at POD1 persisted (Figure 2(d)), with a slight net increase of the particle density in the entire cytoplasm (Figure 2(e)). Staining of the neuropil remained unchanged compared to POD1 (Figure 2(f)). By POD7, MMP-9 particle density in ROI_1_ increased significantly (Figure 2(d)), returning to control levels. Similarly, particle density in the neuropil decreased to return to control levels (Figure 2(f)). No change in particle density was detectable in ROI_2_ at any survival time.

### 3.2. Time Course of MMP-2 Realignments in AVCN Following Cochlear Ablation

Similar to MMP-9, MMP-2 was realigned in AVCN as a consequence of sensory deafferentation. In the normal adult rat, MMP-2 showed a staining pattern reminiscent of that seen with MMP-9. Neuronal cell bodies were strongly labeled, standing out against a faintly labeled neuropil (Figure 1(e)). Compared to MMP-9, MMP-2 responded more sluggishly to sensory deafferentation (Figures 1(f)–1(h)). By POD1, changes in the pattern of MMP-2 staining were barely visible (Figure 1(f)), but the level of MMP-2 in the cytoplasm of neurons had slightly decreased by POD3 (Figure 1(g)). Towards POD7, the decrease grew more pronounced (Figure 1(h)). At that time, neuronal cell bodies were left with low levels of MMP-2 immunoreactivity in their cytoplasm. Notably, MMP-2 staining now revealed beaded collars close to the neuronal plasma membrane (Figure 1(h)), that is, at a time when most such collars visible under MMP-9 staining (Figures 1(b) and 1(c)) had disappeared. As for MMP-9, the overall staining density remained essentially unchanged after cochlear ablation (Figures 1(e)–1(h)). Negative controls of osmicated, flat-embedded sections failed to reveal any staining (Figure 1(k)). 

To test the hypothesis of a redistribution of MMP-2 in AVCN following its sensory deafferentation, the density of MMP-2 immunoreactivity (Figures 2(a)–2(c)) was determined in semithin sections (Figures 2(g)–2(i)). In the AVCN of control animals, MMP-2 was unevenly distributed in the cytoplasm of neurons (Figure 2(g)), much in the same manner as MMP-9. While the majority of MMP-2 immunoreactive particles was located in ROI_1_ near the cell nucleus, the rest was located in ROI_2_ close to the plasma membrane (Figure 2(g)). Following cochlear ablation, particle density of MMP-2 immunoreactivity decreased gradually near the cell nucleus (ROI_1_), whereas it remained on control level in ROI_2_ at all survival times (Figure 2(g)). The gradual decrease of MMP-2 immunoreactivity in cytoplasmatic ROI_1_ from POD1 over POD3 towards POD7 (Figure 2(g)) resulted in an inversion of the ratio of MMP-2 particle densities in cytoplasmatic ROI_1_ and ROI_2_ (Figure 2(g), POD7). Parallel to the net decrease in the cytoplasm of AVCN neurons (Figure 2(h)), the density of MMP-2 positive particles rose progressively in the neuropil towards POD7 (Figure 2(i)), suggesting a reallocation of MMP-2 from intracellular to extracellular realms in AVCN due to the loss of cochlear input.

### 3.3. KA Injections

**Figure 3:**
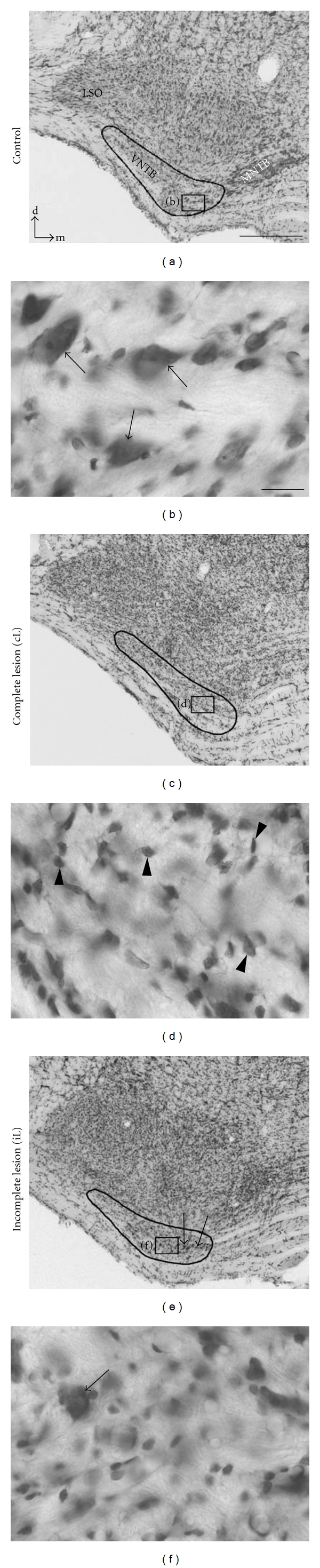
Nissl staining used to document effectiveness and extent of KA injection into VNTB. (a, b) In the superior olivary complex of normal adult rats (a), numerous large neurons (arrows in (b)) were visible in VNTB (outlined in (a), (c), and (e)). Frames seen in (a), (c), and (e) indicate fields shown at high magnification in (b), (d), and (f). Dorsal (d) and medial (m) indicate orientation of brain sections. LSO: lateral superior olive; MNTB: medial nucleus of trapezoid body. (c), (d): VNTB with complete lesion (cL). All neurons have disappeared while glial cells (arrowheads in (d)) increased in number. (e, f) After incomplete VNTB lesions, neurons (arrows in (e) and (f)) survived in parts of VNTB (f). In the case shown in (e), medially residing neurons survived the injection (arrows) while most of the laterally residing neurons disappeared. Scale bar: 50 *μ*m for (a), (c), and (e); 20 *μ*m for (d), (d), and (f).

In order to determine if there are causal dependencies rather than mere coincidences among the deafferentation-dependent expression and redistributions of MMP-9 and MMP-2 on the one hand, and the reinnervation associated with GAP-43 immunoreactivity on the other hand, KA was stereotaxically injected into VNTB prior to cochlear ablation, destroying neuronal cell bodies residing in this nucleus [12]. Nissl staining served to determine success and degree of the excitotoxic lesion. Conspicuously large neuronal cell bodies characterize VNTB (Figures 3(a) and 3(b)). A total loss of neuronal somata in VNTB indicated a complete lesion of VNTB neurons (Figures 3(c) and 3(d)). In several experiments, however, due to an off-center KA injection, neurons were destroyed only in part of VNTB while those in other parts survived the treatment (Figures 3(e) and 3(f)). Control injections of PBS instead of KA into VNTB failed to induce a notable reduction of Nissl-stained cell bodies (not illustrated).

### 3.4. Pattern of GAP-43 Expression Following Cochlear Ablation with or without Preceding VNTB Lesion

**Figure 4:**
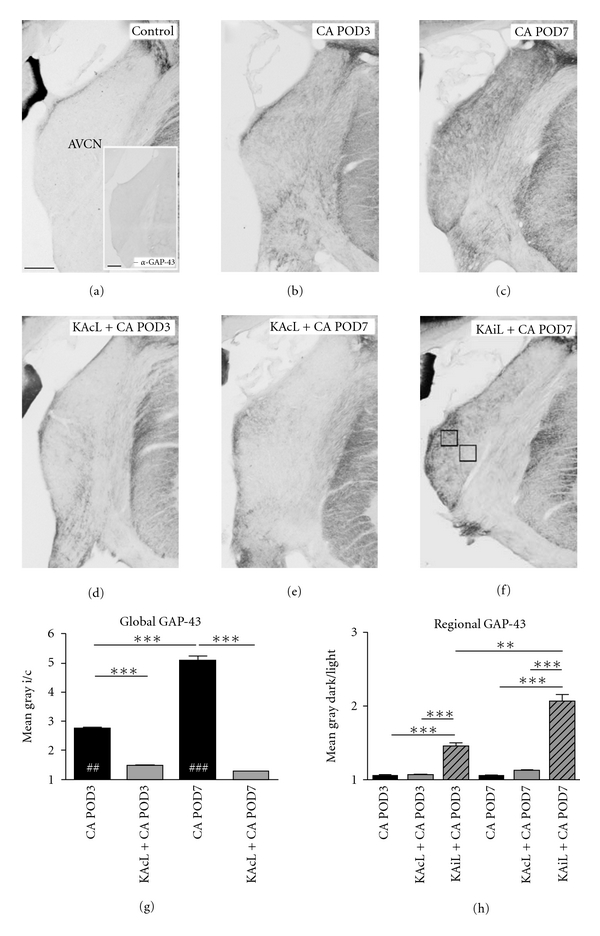
GAP-43 expression in AVCN after complete or incomplete VNTB lesion and subsequent cochlear ablation. (a) Little GAP-43 expression was found in AVCN of control animals. Inset: no staining occurred when omitting the primary antibody. (b, c) Following cochlear ablation, GAP-43 staining emerged around POD3 (b) and reached maximal intensity by POD7 (c). (d, e) Combining complete lesion of VNTB with cochlear ablation (KAcL + CA) led to near-complete failure of GAP-43 expression in AVCN. (f) In cases with incomplete lesion (KAiL + CA) of VNTB, GAP-43 staining failed only locally, producing a patchy staining in AVCN. Frames indicate test fields for quantitative analysis of local GAP-43 staining density. Scale bar in (a)–(f), including inset in (a) 200 *μ*m. Global contrast adjustments are identical for all photographs shown. (g) Staining intensity of GAP-43 immunoreactivity in the AVCN given as gray tone mean. Bars indicate ipsilateral-to-contralateral ratio (i/c) for cases with CA alone and KAcL + CA at POD3 and POD7 following cochlear ablation. Asterisks indicate significant differences among groups, number signs (hash symbols) indicate significant differences against control. (h) Heterogeneity of GAP-43 staining in ipsilateral AVCN after CA, KAcL + CA, and KAiL + CA. Bars indicate ratio between the strongest and faintest GAP-43-stained region within the AVCN.

A healthy rat brainstem is bilaterally symmetric, and staining of tissue in AVCN for any antigen should result in a left-to-right density ratio of close to 1. Neither in normal animals (*P* = 0.41 for left-versus right-staining density) nor in animals receiving a unilateral PBS or KA injection alone (*P* = 0.49 and *P* = 0,45 for left versus right staining density, resp.) did we find an asymmetry of GAP-43 staining in AVCN. In any of these cases, GAP-43 staining was low (Figure 4(a)), confirming a previous study [12]. Negative controls omitting the primary antibodies did not show any staining (Figure 4(a), inset). As a consequence of cochlear ablation, GAP-43 staining emerged in a growing network of fine fibers and boutons and rose significantly in staining density in the ipsilateral AVCN as soon as by POD3 (Figures 4(b) and 4(g), left black bar). By POD7, GAP-43 staining was strong (Figures 4(c) and 4(g), right black bar), with fibers and boutons now prominent. As cochlea and spiral ganglion were always completely ablated in the present study (for incomplete cochlear lesions cp. [5]), GAP-43 immunoreactivity was found in evenly distributed fiber networks throughout the ipsilateral AVCN in Group 3 (Figures 4(b), 4(c), and 4(h), black bars). 

When KA was unilaterally injected to fully destroy VNTB before ablation of the cochlea on the other side, the rise of GAP-43 immunoreactivity failed to occur or was strongly reduced at any time following cochlear lesion (Figures 4(d) and 4(e)). Correspondingly, the ipsilateral-to-contralateral ratio of GAP-43-staining intensity remained close to 1 (Figure 4(g)). When the population of VNTB neurons was incompletely destroyed (Figure 3(e)), a heterogeneous or patchy pattern of GAP-43 staining emerged in AVCN after cochlear ablation (Figure 4(f)), reflecting a partially intact projection from VNTB to AVCN. A patchy distribution instead of an area-wide moderation of GAP-43 immunoreactivity indicated that some topographic order must exist in this projection. A comparison of gray tone means of regions darkly stained for GAP-43 with regions showing less or no GAP-43 staining, both in the AVCN ipsilateral to cochlear ablation, revealed a significant difference (*P* < 0.01 and *P* < 0.001 for POD3 and POD7, resp.; Figure 4(h), shaded bars).

### 3.5. Time Course of MMP-9 Realignment Following Cochlear Ablation with Preceding Complete Lesion of VNTB

**Figure 5:**
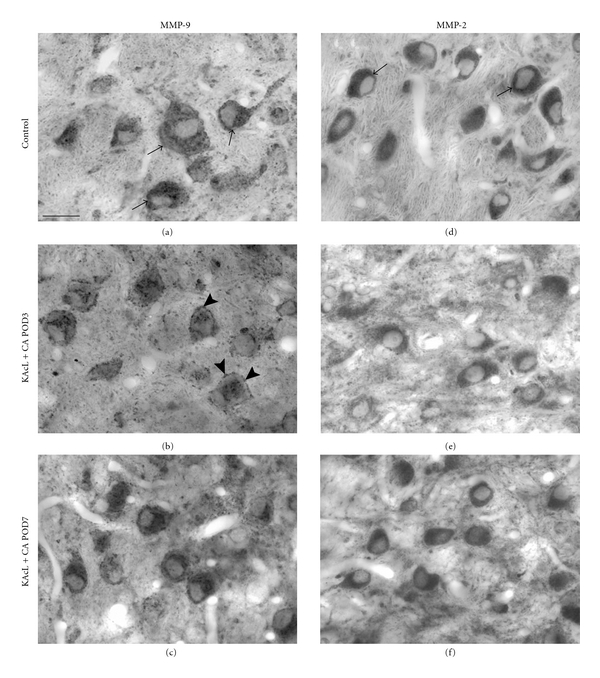
Time course of the distribution of MMP-9 and MMP-2 following combination of KA injection completely lesioning VNTB and cochlear ablation (KAcL + CA). (a)–(c) The distribution of MMP-9 showed no differences compared to the pattern following cochlear ablation alone (Figure 1(a)–1(d)). Compared to control animals (a), the cytoplasm of AVCN neurons (arrows in (a)) have lost MMP-9 by POD3 (b) but regained it till POD7 (c). Beaded collars close to the plasma membrane were particularly prominent around POD3 (arrowheads in (b)). (d–f) As in case with cochlear ablation alone (Figures 1(e)–1(h)), the cytoplasm of AVCN neurons partially lost their MMP-2 immunoreactivity by POD3 (e). By POD7, MMP-2 immunoreactivity in cytoplasm of neurons was almost back to control level (d) again (f). Scale bar: 20 *μ*m. Global contrast adjustment was identical for all photographs in this figure.

When the pattern of MMP-9 immunoreactivity was studied after cochlear ablation preceded by complete destruction of the contralateral VNTB, no differences were observed compared to the expression pattern following cochlear ablation alone (compare Figures 1(c), 1(d) with 5(b), 5(c)). Apparently, axonal growth associated with GAP-43 in AVCN had no effect on the deafferentation-induced development of MMP-9 realignment in AVCN. Neuronal cell bodies that were rich in MMP-9 before cochlear ablation had lost much MMP-9 immunoreactivity by POD3 (Figure 5(b)) but returned to control levels by POD7 (Figure 5(c)) independent of the state of VNTB.

### 3.6. Time Course of MMP-2 Realignment Following Cochlear Ablation with Preceding Complete Lesion of VNTB

By sharp contrast, a preceding lesion of the contralateral VNTB substantially altered the dynamics of MMP-2 actuated by cochlear ablation (Figures 5(d)–5(f)). This change was not yet apparent by POD3 but obvious by POD7. Lesioning VNTB before the cochlea caused MMP-2 expression to increase in the cytoplasm of AVCN neurons at POD7 (Figure 5(f)) to a level that was indistinguishable from control cases (Figure 5(d)), so as if no cochlear ablation had occurred.

### 3.7. Statistical Evaluation of Time Course and Distribution of MMP-9 and MMP-2 Following Cochlear Ablation without or with Preceding Complete Lesion of VNTB

To verify statistical significances of the observations detailed in Sections 3.5 and 3.6, staining densities for MMP-9 and MMP-2 immunoreactivity in the cytoplasm of neurons after cochlear ablation without or with a preceding complete contralateral lesion of VNTB were quantified by measuring gray tone means. Possible variabilities in staining conditions among cases were leveled by calculating the ratio of ipsilateral-to-contralateral staining intensities. 

**Figure 6:**
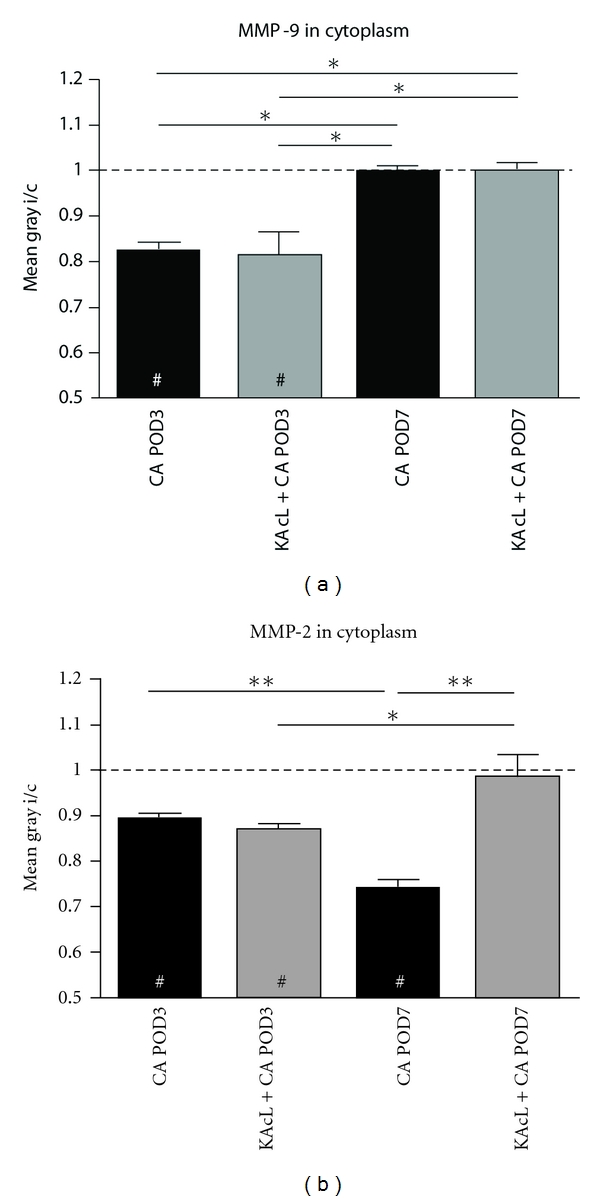
Statistical evaluation of the modulation of MMP-9- and MMP-2-staining intensity in the cytoplasm of AVCN neurons following cochlear ablation (CA) with or without a preceding complete lesion of VNTB (KAcL). (a, b) Bars indicate ipsilateral-to-contralateral ratios (i/c) of gray tone means of MMP-9 (a) and MMP-2 (b) measured in the cytoplasm of AVCN neurons (test field indicated in Figure 1(e)) following cochlear ablation or combination of KA injection with complete lesion of VNTB with cochlear ablation (KAcL + CA). The dashed lines mark a i/c ratio of 1, indicating control level. Whereas VNTB lesions have no influence on the loss and regain of MMP-9 from neuronal cytoplasm after cochlear ablation, MMP-2 levels return to control level by POD7 only if the reinnervation is prevented. Asterisks indicate significant differences among groups, number signs (hash symbols) indicate significant differences against control.

By POD3, MMP-9 staining was significantly lowered compared to controls (Figure 6(a), left bar). This decrease occurred independent of whether VNTB was intact or not (Figure 6(a), second bar). By POD7, MMP-9 staining has regained control levels (Figure 6(a), third bar), again without being influenced by the state of VNTB (Figure 6(a), fourth bar). MMP-2 staining has decreased by POD3 compared to control levels (Figure 6(b), left bar). As for MMP-9 this happened irrespective of the condition of VNTB (Figure 6(b), second bar). Unlike MMP-9, however, MMP-2 staining further decreased towards POD7 after cochlear ablation (Figure 6(b), third bar), provided that the contralateral VNTB was intact. In cases of VNTB lesioning, MMP-2 staining rebounded to control levels (Figure 6(b), fourth bar).

### 3.8. Distribution of MMP-2 in the Ipsilateral AVCN Following Cochlear Ablation with Preceding Incomplete Lesion of VNTB

**Figure 7:**
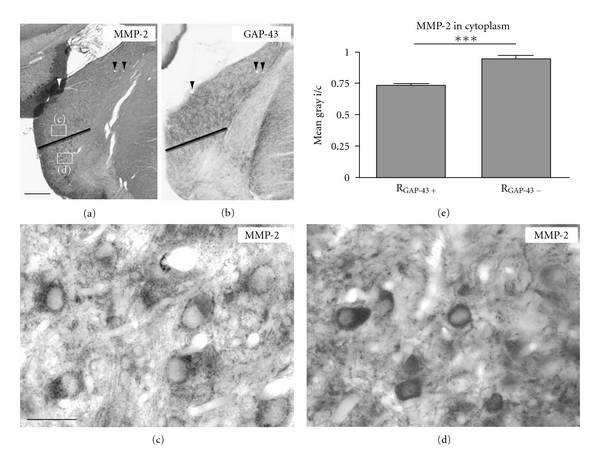
Distribution of MMP-2 in the ipsilateral AVCN on POD7 after incomplete lesion of VNTB due to KA injection followed by cochlear ablation. (a) Section through AVCN stained for MMP-2, showing a distinct border in the staining pattern of MMP-2 (black line). Frames indicate regions shown at higher magnification in (c) and (d). Arrowheads indicate blood vessels also found in the adjacent section shown in (b) stained for GAP-43. (b) In a section through AVCN parallel to the one shown in (a), GAP-43 expression shows a heterogeneity as well (black line) which closely corresponded to the border seen in (a). (c), (d) Photographs at high magnification taken of MMP-2 immunoreactivity in regions of AVCN corresponding to high expression level of GAP-43 (R_GAP−43+_) and low to no expression of GAP-43 ((R_GAP−43−_), respectively. Scale bar: 200 *μ*m for (a), (b); 20 *μ*m for (c), (d). (e) Quantitative evaluation of MMP-2-staining intensity in cytoplasm of local populations of AVCN neurons based on photographs like those shown in (c) and (d).

Cases with an incomplete VNTB lesion opened the possibility for a local analysis of the relationship between MMP-2 realignment and reinnervation as indicated by GAP-43 expression. We noted that the characteristic changes in the pattern of MMP-2 staining seen by 7 days after cochlear ablation occurred only in regions of AVCN where GAP-43 positive fibers have emerged at high density (Figure 7). In favorable cases, a sharp border between GAP-43 rich and GAP-43 poor regions was observed in AVCN (Figure 7(b)). Evaluating staining patterns in pairs of adjacent sections, the heterogeneity of GAP-43 staining was faithfully matched by MMP-2 staining (Figures 7(a), 7(c), and 7(d)). Quantitative analysis (Figure 7(e) revealed a significant difference between the staining intensities of neuronal cytoplasm for MMP-2 in regions corresponding to strong GAP-43 staining (Figure 7(c)) and weak GAP-43 staining (Figure 7(d)).

## 4. Discussion

As a cochlear ablation induces a total sensory deafferentation in the CN, the first central relay of the ascending auditory system, its tissue is reorganized in complex ways. Obviously, a massive degeneration of sensory fibers and their presynaptic endings occurs that has a quick onset and is completed within 10 days [32]. As this happens, reorganization gradually takes over from degeneration. The stages of reorganization encompass different morphological and molecular realignments [11], among them extensive changes to the ECM. We wondered how MMP-9 and MMP-2, key enzymes in ECM modulation, are involved in these processes and how this involvement might be regulated. 

The major findings of the present study are threefold. First, following cochlear ablation, MMP-9 and MMP-2 are realigned in AVCN, and these realignments appeared to be specifically associated with degeneration and reinnervation, respectively. Second, the deafferentation-dependent arrival of GAP-43 positive fibers in AVCN had no effect on changes of MMP-9, neither in the early phase after cochlear ablation when auditory nerve fibers and their synaptic terminals in AVCN degenerate, nor in the subsequent phase of reinnervation when synaptogenesis takes place. Third, level and pattern of MMP-2 were restored to control level by POD7 if reinnervation was prevented. These observations suggest that MMP-9 tends to be associated to neuropil reorganization related to fiber and terminal degeneration, whereas MMP-2 is predominantly involved in assisting reinnervation and synaptogenesis.

### 4.1. Association of MMP-9 and MMP-2 with Tissue Dynamics in Different Postlesional Phases

Our data suggest that following deafferentation of AVCN, overall expression levels of neither MMP-9 nor MMP-2 were changed. Instead, both MMPs were reallocated in numerous AVCN neurons from cytoplasmic regions near the nucleus towards the plasma membrane, and then secreted out into the surrounding neuropil. This we showed quantitatively in osmicated semithin sections. Osmication was done with minimum concentration of osmium and incubation time and therefore did not mask the immunoreactive structures to be analyzed.

Following cochlear ablation, MMP-9 availability and distribution in AVCN were maximally changed as soon as by POD1 and returned to control levels till POD7 (Figures 1(a)–1(d), 2(d)–2(f)). These realignments of MMP-9 as intra- and transcellular migration (Figure 2) occurred early enough to be temporally associated to degenerative events, that is, degeneration of axons and axon terminals as well as oligodendrocytes.

Manipulation of the projection from VNTB to AVCN by KA injection failed to affect the deafferentation-induced development of availability and distribution of MMP-9, indicating independency of MMP-9 from the subsequent reorganization of the neural network texture as indicated by the emergence of GAP-43 positive axons and axon terminals in AVCN. Together with the early onset of changes, the independence of MMP-9 from VNTB integrity and AVCN reinnervation suggest that this metalloproteinase, in the system studied here, acts in the context of degeneration.

MMP-9 function has been implicated in synaptic plasticity and long-term potentiation by extracellular proteolysis [33–36]. However, our data failed to hint at a direct involvement of MMP-9 in synaptogenesis following deafferentation of AVCN due to cochlear ablation. 

The onset of MMP-2 realignment was slightly delayed against that of MMP-9 and proceeded towards POD7 when MMP-9 has already returned to normal. This time course does not match well with degeneration [32] but corresponds temporally to the deafferentation-dependent reinnervation of AVCN by fibers of MOC neurons identified by their contents of GAP-43 (Figures 1(e)–1(h), 2(g)–2(i), and 4(a)–4(c)). When GAP-43 staining level is maximal in the deafferented AVCN, many neuronal somata lost virtually all of their MMP-2 (Figure 1(h)). More importantly, when axons were prevented from reinnervating central parts of AVCN, MMP-2 staining returned back to the predeafferentation pattern by POD7. This indicates a causal dependency of the progression of MMP-2 realignment on the reinnervation of AVCN neurons by GAP-43 containing fibers and boutons.

### 4.2. Consequences of VNTB Lesions on GAP-43 Expression in CN

In spite of a constant volume and concentration of KA injected into VNTB, the radius of the injection site varied considerably among cases, an observation also made for other cerebral systems in which KA was used (e.g., [37]). In the present study we turned this variability to our advantage, analyzing lesions of varying size and position for their systematic consequences on GAP-43 staining in AVCN following cochlear ablation. A complete lesion of VNTB that destroyed all neurons potentially innervating central AVCN following cochlear ablation held GAP-43 expression on control level despite a preceding cochlear ablation. The heterogeneous distribution of GAP-43 immunoreactivity in AVCN following incomplete lesions of VNTB was shown to be related to the survival of large VNTB neurons likely to be MOC neurons projecting to AVCN by axon collaterals [15]. The fact that the surviving subpopulation of VNTB neurons does not raise GAP-43 staining across the entire AVCN but only in distinct regions reveals an independent look on the topographical organization of this projection already suggested by tracing studies [14, 38]. Our findings suggest that laterally positioned neurons project to medial AVCN and medially positioned neurons to lateral AVCN, both on the opposite side of the brainstem.

### 4.3. Contribution of MMP-9 and MMP-2 to Tissue Dynamics in Different Postlesional Phases

We found evidence to relate MMP-9 function in AVCN to the degenerative phase following cochlear ablation. Reeves et al. [39] suggest a role of MMP-9 in the deafferentation/sprouting cycle in hippocampus following unilateral entorhinal cortex lesion. They found that rats receiving an MMP-9 inhibitor failed to develop the capacity for long-term potentiation and showed persistent cellular debris of degenerating terminals not followed by axonal sprouting and synaptogenesis, as was the case in control experiments. Zhang et al. [40] speculate that upregulation of MMP-9 following nerve injury may be part of the tissues' effort to accomplish synaptic remodeling. This is in line with Reeves et al. [39] who showed that no regeneration is initiated if the degenerative phase remains incomplete due to the absence of MMP-9 activity. Contributions of MMP-9 to shape tissue structure following nerve degeneration appear to be part of the prerequisites for reinnervation and synaptogenesis.

Changes in MMP-2 realignment appeared to be underway before the deafferentation-dependent induction of reinnervation of AVCN. Through proteolytic cleavage of receptors, adhesion molecules, and the ECM lattice, attachment sites for new synapses need to be exposed [39, 40]. The onset of MMP-2 secretion before reinnervation of AVCN suggests that MMP-2 mediated remodeling of the pericellular environment prepares the tissue for a reactive synaptogenesis to occur [39, 40].

The essential finding of the present study is that changes in the distribution of MMP-2 after POD3 depend on the presence of GAP-43 containing axons, growth cones, or nascent presynaptic endings, heralding reinnervation including synaptogenesis. It is known that MMPs are involved in axonal guidance. For instance, broad-spectrum metalloproteinase inhibitors applied *in vivo* to retinal ganglion cells led to misguided axons in the developing visual system [41]. MMPs have been implicated in modulating the interaction of growth cone guidance cues with their receptors through cleavage of their ectodomains [42–44]. Future directions to test the crucial role of MMP-2 in axonal growth and synaptogenesis could be experiments including specific MMP-2 inhibitors or MMP-2 knockouts. 

The observations made in our study are consistent with reports on the implication of MMP-2 in synaptogenesis and synapse remodeling through cleavage of ECM proteins like laminin [45] and brevican [46], as well as growth factors like TNF-*α* [47], and brain derived neurotrophic factor [48], all of which have profound effects on synaptic plasticity and synaptogenesis (for review see [25]). Eventually, there seems to be a reciprocal relationship between induction of axonal growth and reactive synaptogenesis on the one hand and the reshuffling of MMP-2 on the other. Whereas induction of reinnervation turned out to be necessary to maintain the MMP-2 response over the phase of AVCN reinnervation, MMP-2 appears to facilitate GAP-43-directed axonal growth and synaptogenesis by molding ECM molecules, allowing growing axons to permeate the tissue and find their targets. 

A differential functional association of MMP-9 and MMP-2 is also suggested by the type and the temporal succession of specific immunoreactive structures. Collars of particles spatially associated to the plasma membrane of neuronal cell bodies in AVCN were seen to develop after deafferentation by use of antibodies either raised against MMP-9 (Figures 1(b) and 1(c)) or MMP-2 (Figure 1(h)). These beaded collars were reminiscent of synaptophysin-stained collars known to reveal rings of presynaptic endings [4]. When deafferentation caused many presynaptic endings to perish [49], MMP-9 positive collars arose quickly, suggesting action related to degradation of decaying presynaptic endings. By contrast, MMP-2-positive collars were present by POD7 when MMP-9 collars had already disappeared (Figures 1(d), 1(h)). By that time, neurons of AVCN have developed collars of GAP-43-positive presynaptic endings [4, 10]. This suggests action of MMP-2 specifically related to the formation of new synaptic contacts. The absence of MMP-2-positive collars in cases when synaptogenesis in AVCN is prevented by VNTB lesion lends strong support to this reading of our observations.

Perineuronal nets are specializations of the ECM that enwrap neuronal cell bodies, serving to resist structural changes to existing synaptic contacts [50, 51]. Among the neuronal populations bearing perineuronal nets are neurons of the AVCN [52–54]. Foscarin et al. [55] noted a rise of activity of MMP-9 or MMP-2 or both with decreasing integrity of perineuronal nets. This faltering of perineuronal nets was induced in the deep cerebellar nuclei of mice that were exposed to enriched environments, over-expressing GAP-43 in cerebellar Purkinje cells. Again, these observations do not exclude the possibility that MMP-9 and MMP-2 differentially contribute to synaptogenesis, the one perhaps preparatory, the other aiding, that takes place in the adult mammalian brain upon nerve injury or leaning.

### 4.4. Conclusions

Our data suggest MMP-9 and MMP-2 to move out of neuronal cell bodies of the AVCN following its total sensory deafferentation by cochlear ablation. We showed that the reinnervation taking place after auditory nerve degeneration is essential to maintain and promote changes in distribution and availability of MMP-2, but not of MMP-9. In our experimental model, MMP-9 function is straightforwardly attributable to nerve and synapse degradation following cochlear ablation, whereas MMP-2, preactivated in AVCN to prepare for reinnervation, is functionally attributable to a compensatory reorganization of the deafferented neuronal network that includes axonal sprouting and synaptogenesis.

## Figures and Tables

**Table 1:** Experimental groups.

Group	Surgical procedures	POD following cochlear ablation	Number of brains	Immunohistochemical procedures
1	—	—	5	GAP-43 ABC-DAB MMP-2 ABC-DAB + flat-embedding + semithin sectioning MMP-9 ABC-DAB + flat-embedding + semithin sectioning
2	PBS injection into VNTB → recovery for 1–3 weeks	—	2	GAP-43 ABC-DAB MMP-2 ABC-DAB + flat-embedding MMP-9 ABC-DAB + flat-embedding
	KA injection into VNTB → recovery for 1–3 weeks	—	2	GAP-43 ABC-DAB MMP-2 ABC-DAB + flat-embedding MMP-9 ABC-DAB + flat-embedding
3	Cochlear ablation	POD1 POD3 POD7	4 3 5	GAP-43 ABC-DAB MMP-2 ABC-DAB + flat-embedding + semithin sectioning MMP-9 ABC-DAB + flat-embedding + semithin sectioning
4	KA injection into VNTB → recovery for 1-3 weeks → cochlear ablation	POD3 POD7	3 cL/2 iL 5 cL/3 iL	GAP-43 ABC-DAB MMP-2 ABC-DAB + flat-embedding MMP-9 ABC-DAB + flat-embedding

cL: complete lesion of VNTB; DAB: diaminobenzidine staining; iL: incomplete lesion of VNTB; KA: kainic acid; PBS: phosphate buffered saline; POD: postoperative day.

**Table 2:** Antibodies.

Antigen	Immunogen	Source	Working dilution
Primary Antibodies			

Growth associated protein 43 (GAP-43), clone 9-1E12	43-48 kDa GAP-43 (B-50, F1 or pp46) purified from rat brain.	Millipore, Temecula, Calif, USA; mouse, monoclonal, IgG_1_, cat.no. MAB347.	1 : 5,000
Matrix metalloproteinase 2 (MMP-2), clone H-76	raised against amino acids 1–76 of MMP-2 of human origin.	Santa Cruz Biotechnology, Inc. Santa Cruz, Calif, USA; rabbit, polyclonal, IgG, cat.no. sc-10736.	1 : 100
Matrix metalloproteinase 9 (MMP-9) clone C-20	raised against a peptide mapping at the C-terminus of MMP-9 of human origin.	Santa Cruz Biotechnology, Inc., Santa Cruz, Calif, USA; goat, polyclonal, IgG, cat.no. sc-6840.	1 : 250

Secondary Antibodies			

Horse anti-mouse biotinylated	Mouse IgG	Vector laboratories	1 : 200
Goat anti-rabbit biotinylated	Rabbit IgG	Vector laboratories	1 : 200
Rabbit anti-goat biotinylated	Goat IgG	Vector laboratories	1 : 200

## Abbreviations

*α*: “Anti,” specificity of primary antibody
ABC: Avidin-biotin complex
ANOVA: Analysis of variance
AVCN: Anteroventral cochlear nucleus
c: Contralateral
CA: Cochlear ablation
cL: Complete KA lesion of VNTB
CN: Cochlear nucleus
CNS: Central nervous system
ctrl: Control
d: Dorsal
DAB: 3.3-diaminobenzidine tetrahydrochloride
ECM: Extracellular matrix
GAP-43: Growth associated protein 43
i: Ipsilateral
i/c: Ipsilateral-to-contralateral ratio
iL: Incomplete KA lesion of VNTB
KA: Kainic acid
l/r: Left-to-right ratio
LSO: Lateral superior olive
m: Medial
MMP: Matrix metalloproteinase
MNTB: Medial nucleus of the trapezoid body
MOC: Medial olivocochlear
ns: Not significant
nu: Nucleus
PBS: Phosphate-buffered saline
POD: Postoperative day
ROI: Region of interest
VNTB: Ventral nucleus of the trapezoid body.

## References

[1] Kane EC (1974). Patterns of degeneration in the caudal cochlear nucleus of the cat after cochlear ablation. *Anatomical Record*.

[2] Oliver DL, Potashner SJ, Jones DR, Morest DK (1983). Selective labeling of spiral ganglion and granule cells with D-asparatate in the auditory system of cat and guinea pig. *Journal of Neuroscience*.

[3] Altschuler RA, Sheridan CE, Horn JW, Wenthold RJ (1989). Immunocytochemical localization of glutamate immunoreactivity in the guinea pig cochlea. *Hearing Research*.

[4] Illing R-B, Horváth M, Laszig R (1997). Plasticity of the auditory brainstem: effects of cochlear ablation on GAP-43 immunoreactivity in the rat. *Journal of Comparative Neurology*.

[5] Illing R-B, Kraus KS, Meidinger MA (2005). Reconnecting neuronal networks in the auditory brainstem following unilateral deafening. *Hearing Research*.

[6] Benowitz LI, Routtenberg A (1997). GAP-43: an intrinsic determinant of neuronal development and plasticity. *Trends in Neurosciences*.

[7] Horváth M, Förster CR, Illing R-B (1997). Postnatal development of GAP-43 immunoreactivity in the auditory brainstem of the rat. *Journal of Comparative Neurology*.

[8] de la Monte SM, Federoff HJ, Ng SC, Grabczyk E, Fishman MC (1989). GAP-43 gene expression during development: persistence in a distinctive set of neurons in the mature central nervous system. *Developmental Brain Research*.

[9] Illing R-B, Horváth M (1995). Re-emergence of GAP-43 in cochlear nucleus and superior olive following cochlear ablation in the rat. *Neuroscience Letters*.

[10] Meidinger MA, Hildebrandt-Schoenfeld H, Illing R-B (2006). Cochlear damage induces GAP-43 expression in cholinergic synapses of the cochlear nucleus in the adult rat: a light and electron microscopic study. *European Journal of Neuroscience*.

[11] Illing R-B, Rosskothen-Kuhl N, Fredrich M, Hildebrandt H, Zeber AC (2010). Imaging the plasticity of the central auditory system on the cellular and molecular level. *Audiological Medicine*.

[12] Kraus KS, Illing R-B (2004). Superior olivary contributions to auditory system plasticity: medial but not lateral olivocochlear neurons are the source of cochleotomy-induced GAP-43 expression in the ventral cochlear nucleus. *Journal of Comparative Neurology*.

[13] White JS, Warr WB (1983). The dual origins of the olivocochlear bundle in the albino rat. *Journal of Comparative Neurology*.

[14] Brown MC, Pierce S, Berglund AM (1991). Cochlear-nucleus branches of thick (medial) olivocochlear fibers in the mouse: a cochleotopic projection. *Journal of Comparative Neurology*.

[15] Horváth M, Kraus KS, Illing R-B (2000). Olivocochlear neurons sending axon collaterals into the ventral cochlear nucleus of the rat. *Journal of Comparative Neurology*.

[16] Brown MC, Liberman MC, Benson TE, Ryugo DK (1988). Brainstem branches from olivocochlear axons in cats and rodents. *Journal of Comparative Neurology*.

[17] Ryan AF, Keithley EM, Wang ZX, Schwartz IR (1990). Collaterals from lateral and medial olivocochlear efferent neurons innervate different regions of the cochlear nucleus and adjacent brainstem. *Journal of Comparative Neurology*.

[18] Michaluk P, Kaczmarek L (2007). Matrix metalloproteinase-9 in glutamate-dependent adult brain function and dysfunction. *Cell Death and Differentiation*.

[19] Amantea D, Corasaniti MT, Mercuri NB, Bernardi G, Bagetta G (2008). Brain regional and cellular localization of gelatinase activity in rat that have undergone transient middle cerebral artery occlusion. *Neuroscience*.

[20] Yang Y, Candelario-Jalil E, Thompson JF (2010). Increased intranuclear matrix metalloproteinase activity in neurons interferes with oxidative DNA repair in focal cerebral ischemia. *Journal of Neurochemistry*.

[21] Yong VW (2005). Metalloproteinases: mediators of pathology and regeneration in the CNS. *Nature Reviews Neuroscience*.

[22] Milward EA, Fitzsimmons C, Szklarczyk A, Conant K (2007). The matrix metalloproteinases and CNS plasticity: an overview. *Journal of Neuroimmunology*.

[23] Pizzi MA, Crowe MJ (2007). Matrix metalloproteinases and proteoglycans in axonal regeneration. *Experimental Neurology*.

[24] Sternlicht MD, Werb Z (2001). How matrix metalloproteinases regulate cell behavior. *Annual Review of Cell and Developmental Biology*.

[25] Ethell IM, Ethell DW (2007). Matrix metalloproteinases in brain development and remodeling: synaptic functions and targets. *Journal of Neuroscience Research*.

[26] Fredrich M, Illing R-B (2010). MMP-2 is involved in synaptic remodeling after cochlear lesion. *NeuroReport*.

[27] Rivera S, Ogier C, Jourquin J (2002). Gelatinase B and TIMP-1 are regulated in a cell- and time-dependent manner in association with neuronal death and glial reactivity after global forebrain ischemia. *European Journal of Neuroscience*.

[28] Costanzo RM, Perrino LA, Kobayashi M (2006). Response of matrix metalloproteinase-9 to olfactory nerve injury. *NeuroReport*.

[29] Ranasinghe HS, Williams CE, Christophidis LJ, Mitchell MD, Fraser M, Scheepens A (2009). Proteolytic activity during cortical development is distinct from that involved in hypoxic ischemic injury. *Neuroscience*.

[30] Hsu JYC, Bourguignon LYW, Adams CM (2008). Matrix metalloproteinase-9 facilitates glial scar formation in the injured spinal cord. *Journal of Neuroscience*.

[31] Paxinos G, Watson C (1986). *The Rat Brain in Stereotaxic Coordinates*.

[32] Gentschev T, Sotelo C (1973). Degenerative patterns in the ventral cochlear nucleus of the rat after primary deafferentation. An ultrastructural study. *Brain Research*.

[33] Szklarczyk A, Lapinska J, Rylski M, McKay RDG, Kaczmarek L (2002). Matrix metalloproteinase-9 undergoes expression and activation during dendritic remodeling in adult hippocampus. *Journal of Neuroscience*.

[34] Nagy V, Bozdagi O, Matynia A (2006). Matrix metalloproteinase-9 is required for hippocampal late-phase long-term potentiation and memory. *Journal of Neuroscience*.

[35] Bozdagi O, Nagy V, Kwei KT, Huntley GW (2007). In vivo roles for matrix metalloproteinase-9 in mature hippocampal synaptic physiology and plasticity. *Journal of Neurophysiology*.

[36] Oliveira-Silva P, Jurgilas PB, Trindade P (2007). Matrix metalloproteinase-9 is involved in the development and plasticity of retinotectal projections in rats. *NeuroImmunoModulation*.

[37] Gaddy JR, Britt MD, Neill DB, Haigler HJ (1979). Susceptibility of rat neostriatum to damage by kainic acid: age dependence. *Brain Research*.

[38] Spangler KM, Cant NB, Henkel CK, Farley GR, Warr WB (1987). Descending projections from the superior olivary complex to the cochlear nucleus of the cat. *Journal of Comparative Neurology*.

[39] Reeves TM, Prins ML, Zhu J, Povlishock JT, Phillips LL (2003). Matrix metalloproteinase inhibition alters functional and structural correlates of deafferentation-induced sprouting in the dentate gyrus. *Journal of Neuroscience*.

[40] Zhang X, Cheng M, Chintala SK (2004). Kainic acid-mediated upregulation of matrix metalloproteinase-9 promotes retinal degeneration. *Investigative Ophthalmology and Visual Science*.

[41] Webber CA, Hocking JC, Yong VW, Stange CL, McFarlane S (2002). Metalloproteases and guidance of retinal axons in the developing visual system. *Journal of Neuroscience*.

[42] Zuo J, Ferguson TA, Hernandez YJ, Stetler-Stevenson WG, Muir D (1998). Neuronal matrix metalloproteinase-2 degrades and inactivates a neurite- inhibiting chondroitin sulfate proteoglycan. *Journal of Neuroscience*.

[43] Galko MJ, Tessier-Lavigne M (2000). Function of an axonal chemoattractant modulated by metalloprotease activity. *Science*.

[44] Hattori M, Osterfield M, Flanagan JG (2000). Regulated cleavage of a contact-mediated axon repellent. *Science*.

[45] Thrailkill KM, Quarles LD, Nagase H, Suzuki K, Serra DM, Fowlkes JL (1995). Characterization of insulin-like growth factor-binding protein 5-degrading proteases produced throughout murine osteoblast differentiation. *Endocrinology*.

[46] Nakamura H, Fujii Y, Inoki I (2000). Brevican is degraded by matrix metalloproteinases and aggrecanase-1 (ADAMTS4) at different sites. *Journal of Biological Chemistry*.

[47] Gearing AJH, Beckett P, Christodoulou M (1994). Processing of tumour necrosis factor-*α* precursor by metalloproteinases. *Nature*.

[48] Jung JH, Park MH, Choi SY, Koh JY (2005). Activation of the Trk signaling pathway by extracellular zinc. Role of metalloproteinases. *Journal of Biological Chemistry*.

[49] Hildebrandt H, Hoffmann NA, Illing R-B (2011). Synaptic reorganization in the adult rat's ventral cochlear nucleus following its total sensory deafferentation. *PLoS ONE*.

[50] Pizzorusso T, Medini P, Berardi N, Chierzi S, Fawcett JW, Maffei L (2002). Reactivation of ocular dominance plasticity in the adult visual cortex. *Science*.

[51] Carulli D, Pizzorusso T, Kwok JCF (2010). Animals lacking link protein have attenuated perineuronal nets and persistent plasticity. *Brain*.

[52] Cant NB, Benson CG (2006). Wisteria floribunda lectin is associated with specific cell types in the ventral cochlear nucleus of the gerbil, Meriones unguiculatus. *Hearing Research*.

[53] Hilbig H, Nowack S, Boeckler K, Bidmon H-J, Zilles K (2007). Characterization of neuronal subsets surrounded by perineuronal nets in the rhesus auditory brainstem. *Journal of Anatomy*.

[54] Wagoner JL, Kulesza RJ (2009). Topographical and cellular distribution of perineuronal nets in the human cochlear nucleus. *Hearing Research*.

[55] Foscarin S, Ponchione D, Pajaj E (2011). Experience-dependent plasticity and modulation of growth regulatory molecules at central synapses. *PLoS ONE*.

